# Spanish People with Type 2 Diabetes Show an Improved Adherence to the Mediterranean Diet

**DOI:** 10.3390/nu12020560

**Published:** 2020-02-20

**Authors:** Nuria Alcubierre, Minerva Granado-Casas, Jordi Real, Hèctor Perpiñán, Esther Rubinat, Mireia Falguera, Esmeralda Castelblanco, Josep Franch-Nadal, Didac Mauricio

**Affiliations:** 1 Department of Nutrition and Dietetics, Avantmedic, 25008 Lleida, Spain; nurialcubierre@gmail.com; 2 Department of Endocrinology and Nutrition, Health Sciences Research Institute & University Hospital Germans Trias i Pujol, 08916 Badalona, Spain; mgranado@igtp.cat; 3 Lleida Institute for Biomedical Research Dr. Pifarré Foundation IRB Lleida, University of Lleida, 25198 Lleida, Spain; rubinatesther@gmail.com; 4 DAP-Cat Group, Unitat de Suport a la Recerca de Barcelona, Institut Universitari d’Investigació en Atenció Primària Jordi Gol (IDIAP Jordi Gol), 08007 Barcelona, Spain; jordi.real@gmail.com; 5 Center for Biomedical Research on Diabetes and Associated Metabolic Diseases (CIBERDEM), Instituto de Salud Carlos III, 08907 Barcelona, Spain; esmeraldacas@gmail.com; 6 Conselleria de Sanitat Universal i Salut Pública, Generalitat Valenciana, 46010 Valencia, Spain; perpinan_hec@gva.es; 7 Department of Nursing and Physiotherapy, Serra Hunter Lecture, University of Lleida, 25198 Lleida, Spain; 8 Primary Health Care Center Igualada Nord, 08700 Igualada, Spain; mireiafalguera@hotmail.com; 9 Department of Endocrinology and Nutrition, Hospital de la Santa Creu i Sant Pau & Sant Pau Biomedical Research Institute (IIB Sant Pau), 08041 Barcelona, Spain; 10 Department of Medicine, Barcelona Autonomous University (UAB), 08035 Barcelona, Spain

**Keywords:** type 2 diabetes, dietary quality index, medical nutrition therapy, dietary pattern, Mediterranean diet, healthy eating

## Abstract

The aim of this study was to assess the dietary pattern (i.e., Mediterranean Diet (MedDiet) and healthy eating) in people with type 2 diabetes (T2D) compared with those without diabetes. In addition, we explored clinical factors associated with the dietary pattern. This cross-sectional study was performed with a sample of 476 participants (238 with T2D and 238 participants without diabetes, matched for age and sex). The alternate Mediterranean Diet (aMED) score and the alternate Healthy Eating Index (aHEI) were calculated. Statistical analysis included comparison between groups and multivariable models. Participants with T2D showed higher aMED and aHEI scores (mean (SD): 4.3 (1.5) and 43.9 (6.5), respectively) in comparison with the control group (3.5 (1.8) and 39.4 (7.4), respectively; *p* < 0.001). In addition, a higher proportion of participants with T2D in higher tertiles of aMED (21.8%) and aHEI (39.9%) was observed compared with participants without diabetes (11.3% for the aMED, and 19.3% for the aHEI; *p* < 0.001). The adjusted multivariable analysis revealed that T2D (*p* *<* 0.001), increasing age (*p* *=* 0.006 and *p* = 0.030, respectively), and physical activity (*p* *=* 0.009) were positively associated with higher aMED and aHEI scores. Dyslipidemia and female gender were positively associated with aMED and aHEI (*p* = 0.031 and *p* < 0.001, respectively). The specific multivariable analysis for the group with T2D yielded a positive association of age (*p* < 0.001) and dyslipidemia (*p* = 0.021) with aMED. Regarding the aHEI, only female gender was positively related with this score in diabetes participants (*p* = 0.025). Participants with T2D showed a higher adherence to the MedDiet and a healthier eating pattern.

## 1. Introduction

The American Diabetes Association (ADA) has established that lifestyle management is a key element in the treatment of people with diabetes to ensure an optimal control of the disease [1]. This includes diabetes self-management education and support (DSMES), medical nutrition therapy (MNT), physical activity, smoking cessation counseling, and psychosocial care. DSMES provides people with diabetes with the essential tools to adopt self-management decisions; furthermore, DSMES has been shown to be associated with optimal management of the disease, lowering glycated hemoglobin (HbA1c) and body weight, reducing all-cause mortality risk and improving the quality of life of this population [1]. Although a standardized dietary pattern for people with diabetes does not exist, the Mediterranean Diet (MedDiet) has many benefits in the prevention and treatment of type 2 diabetes mellitus (T2D) [1,2,3,4,5]. Furthermore, a sub-analysis of the PREDIMED study, a multicenter randomized controlled trial designed to assess the effect of the MedDiet on cardiovascular outcomes in individuals at high cardiovascular risk, found that the MedDiet, supplemented with virgin olive oil or nuts, showed a reduction of 52% in the incidence of T2D [6]. Moreover, the Nurse’s Health Study and Health Professionals Follow-up Study found an 11% reduction in T2D risk associated with a healthier eating pattern [7].

Few studies have so far assessed the efficacy of the DSMES, including the adherence to the healthy eating pattern and the MedDiet in people with T2D [8,9,10,11,12]. Only one cross-sectional study performed on a small sample of participants (*n* = 65 T2D and *n* = 46 non-diabetic controls) assessed the dietary pattern (i.e. MedDiet and healthy eating) in both groups using the Mediterranean Diet score (MED), the alternate Mediterranean Diet (aMED) score, the Healthy Eating Index (HEI), and the alternate Healthy Eating Index (aHEI) [9]. They found lower MED, aMED, and HEI scores in participants with T2D compared with the controls, without differences in the aHEI between both groups. Huffman et al. performed a cross-sectional study to assess the healthy eating pattern among African Americans and Haitians with and without T2D, and also found a lower adherence to the healthy eating pattern in those with diabetes [8]. In addition, Vidal-Peracho et al. found a higher adherence to the MedDiet in a group without diabetes compared with participants with both types of diabetes (type 1 and type 2) [10]. Two post-hoc analyses of prospective studies assessed the relationship between the MedDiet and healthy eating with cardiovascular risk in participants with T2D without a control group [11,12]; they found an inverse association between cardiovascular risk factors, the MedDiet, and healthy eating patterns. In addition, other studies performed in subjects with T2D have only assessed the nutrient intake and food consumption of this population compared with nutritional recommendations, without assessing the dietary pattern [13,14,15,16]. Among these, two cross-sectional studies observed a low adherence to the nutritional recommendations in participants with T2D [13,16]. However, another study observed an improved adherence to the nutritional recommendations, except for saturated fatty acid (SFA) intake, in participants with T2D [15]. Finally, an investigation using two multicenter prospective studies with large cohorts, the European Prospective Investigation into Cancer and Nutrition (EPIC) study and the Multiethnic Cohort Study (MEC), showed that, compared to those without diabetes, participants with diabetes (both type 1 and type 2) showed little difference in the consumption of soft drinks, sweets, juice, vegetables, fish, meat, wine, and beer [14].

To the best of our knowledge, this is the first study to assess differences in the dietary pattern (i.e., MedDiet and healthy eating) between participants with T2D and a group without diabetes, including the daily nutrient intake and food consumption. The aMED and aHEI have been strongly correlated with specific biomarkers involved in the development of chronic diseases, such as diabetes [17]. Both scores are focused on dietary patterns rich in fruits and vegetables, whole grains, nuts, fish, and moderate alcohol consumption; for this reason, these two scores are useful measures of dietary patterns and their associated cardiovascular risk [17,18,19]. In our Mediterranean country, people with T2D usually receive DSMES that includes MNT focused on the MedDiet and healthy eating patterns. Therefore, we hypothesized that people with T2D would show a higher adherence to the MedDiet and a healthier eating pattern in comparison with people without diabetes. The aim of the study was to assess differences in the dietary pattern (i.e., MedDiet and healthy eating) between participants with T2D and those without diabetes. Additionally, clinical factors associated with the dietary pattern were explored.

## 2. Materials and Methods

### 2.1. Study Design

We used a cross-sectional study design. A total sample of 238 participants with T2D and 238 sex- and age-matched participants without diabetes were recruited. This was a sub-study using the cohorts of two previously published studies [20,21]: the first one included a cohort of subjects with T2D designed to assess their quality of life and treatment satisfaction; the second study was a population-based cohort study designed to assess the prevalence of prediabetes, and risk factors associated with this condition. Participants with T2D were recruited at the University Hospital Arnau de Vilanova in Lleida (Spain), the reference specialized hospital for the province of Lleida. Non-diabetic individuals were randomly selected from the population-based study from the same area to match the age and sex distribution of the T2D group. In the T2D group, the inclusion criteria were as follows: diagnosis of T2D, and age between 40 and 75 years. The exclusion criteria were as follows: being a healthcare professional, participants who showed cognitive deterioration, such as dementia and mental diseases; presence of macrovascular complications (i.e., heart failure, cerebrovascular disease, ischemic heart disease, and peripheral arterial disease), previous diabetic foot disease, macroalbuminuria (defined as a urine albumin/creatinine ratio over 300 mcg/g), renal failure (defined as a glomerular filtration rate < 60 mL/min), and the presence of any condition that required specific MNT (e.g., pregnancy). In the group without diabetes, the inclusion criteria were participants aged over 25 years and belonging to the specific primary health care area. The exclusion criteria were the same as in the T2D group, together with the diagnosis of diabetes. More details on the characteristics of the two study groups are described in the previous publications [20,21]. The recruitment of both studies occurred between March 2010 and July 2014. The Ethics committee of the University Hospital Arnau de Vilanova of Lleida (CEIC 1079) approved both studies. Written informed consent was obtained from each of the study participants.

### 2.2. Clinical Variables

Medical records were thoroughly reviewed to collect clinical and sociodemographic data. Blood samples and anthropometric variables were collected by standardized methods; these were detailed in previous publications [20,21]. Hypertension and dyslipidemia were defined as receiving medication for these given conditions (i.e. antihypertension and lipid-lowering drugs, respectively). Physical activity was determined using the validated method of Bernstein et al. [22]; this was classified as sedentary if the expenditure energy was <10% of the daily energy expenditure from performing any physical activity that requires at least 4 METS (The Metabolic Equivalents; i.e., walking or cycling for more than 25 min/day), and as regular physical activity when the participant spent >10% of the expenditure energy with any of these activities. According to the World Health Organization (WHO), one MET is defined as the energy cost of sitting quietly and is equivalent to a caloric consumption of 1 Kcal/Kg/h [23]. Educational level was classified as no university if the participant did not have a university degree, or graduate, or higher if he/she had a university education. Tobacco exposure included current and former smokers.

### 2.3. Dietary Assessment

The food frequency questionnaire (FFQ) designed by Willet et al., adapted and validated for a Spanish population, was individually administered to each of the study participants [24,25,26]. This is a semiquantitative questionnaire based on the Nurse’s Health Study and used in our previous publications [27]. This 101-item questionnaire collects data on food consumption over the previous year prior to the visit [28]. Moreover, this FFQ has been used to estimate the nutritional intake and food consumption up to a period of 6 years, with demonstrated good reproducibility [26]. More details of this FFQ were described in a previous publication [26].

The dietary pattern was assessed using two dietary quality indexes (i.e., aMED and aHEI) [17,18,19]. The aMED includes nine key components of the traditional MedDiet: fruits (including nuts and seeds), vegetables (not including potatoes), legumes, cereals (only whole grain), dairy products, fish, meat (not including poultry), monounsaturated-to-SFA ratio and alcohol consumption. One point was given to the components that are typically representative of the MedDiet (legumes, fruits, vegetables, cereals, and fish). This score ranges from 0 (minimal adherence) to 9 (maximal adherence) [17,19]. The aHEI was calculated according to the criteria of McCullough et al. [18]; this index includes vegetables, fruit, nuts and soy, meat (white and red), cereal fiber, trans fat, polyunsaturated and saturated fat, and alcohol consumption. The score was determined excluding long-term multivitamin use, with ranges from 0 (low quality diet) to 80 (high quality diet).

Nutrient values were obtained from the food composition tables of the US Department of Agriculture and other published sources for Spanish and English foods and portion sizes [29,30,31]. In addition, we used the Spanish food composition tables to avoid an overestimation of nutrient intake from the fortified dairy products in the US. Finally, daily food consumption and nutrient intake were adjusted for energy intake.

### 2.4. Statistical Analysis

Descriptive analysis between both groups (T2D and non-diabetes) was performed. The quantitative variables were described using the mean and standard deviation (SD) or the median and interquartile range, according to the normal and non-normal distributions. Categorical variables were summarized using the absolute (*n*) and relative (%) frequencies. The dietary quality indexes were classified divided in tertiles, from T1 (low adherence) to T3 (high adherence). Statistical significance was determined using the Chi-squared test to assess the differences between the frequencies. The t-Student test was performed to assess the differences between quantitative variables. The method of Benjamini and Hochberg (“BH” and “BY”) was performed to control the false discovery rate and the expected proportion of false discoveries among the rejected hypothesis [32]. The relationship between both groups in terms of dietary quality indexes was assessed with multivariable logistic models adjusting for potential confounders (these were variables statistically significant in the bivariate analysis or clinically associated with diabetes). The multivariable logistic models were designed to explain the higher tertiles of aMED and aHEI. The goodness-of-fit assumption using the Hosmer–Lemeshow test for logistic models was assessed. The odds ratio (OR) with 95% confidence interval (95% CI) were used to estimate the measures. A *p* < 0.05 was considered statistically significant for all the tests.

## 3. Results

Clinical and sociodemographic characteristics of both study groups are shown in Table 1. Participants with T2D had a higher body mass index (BMI) (*p <* 0.001), waist circumference (*p <* 0.001), glycated hemoglobin (HbA1c) (*p <* 0.001), and triglyceride concentration (*p* < 0.001) in comparison with the control group. Moreover, participants with T2D were more sedentary than the controls (49.8% vs. 70.5% for regular physical activity; *p <* 0.001). Furthermore, participants with T2D showed a higher frequency of hypertension and dyslipidemia (*p* < 0.001 and *p* = 0.001, respectively). Despite total cholesterol and LDL-cholesterol being lower in participants with T2D, triglyceride concentration was higher in this study group (*p <* 0.001); HDL-cholesterol was also lower in participants with T2D (*p* < 0.001).

### 3.1. Dietary Pattern

The adherence to the MedDiet and healthy eating patterns (measured with the aMED and aHEI, respectively) are shown in Table 2. Participants with T2D had a higher adherence to the MedDiet and a healthier eating pattern with higher aMED and aHEI scores (mean (SD): 4.3 (1.5) and 43.9 (6.5), respectively) in comparison with the control group (3.5 (1.8) and 39.4 (7.4), respectively; *p* < 0.001). Moreover, there was a higher proportion of participants with T2D in higher tertiles of aMED (21.8% for T2D vs. 11.3% for controls, *p* < 0.001) and aHEI (39.9% for T2D vs. 19.3% for controls; *p* < 0.001). These differences between the groups are the result of specific differences in terms of daily food consumption and nutrient intake (Table A1 and Table A2, respectively). Participants with T2D showed a higher intake of dairy products (*p* = 0.011), white meat (*p* = 0.002), lean fish (*p* = 0.002), and fish (*p* = 0.039) (Table A1); furthermore, T2D participants had a lower intake of bread (*p* = 0.016) and salt (*p <* 0.001) in comparison with the control group. On the other hand, participants with T2D showed a higher intake of prepared meals (*p* = 0.009) and non-alcoholic beverages (*p* = 0.011) than the controls.

In terms of daily nutrient intake, as evaluated using the FFQ (Table A2), T2D participants had a lower glycemic load (*p =* 0.034) and a lower intake of complex carbohydrate (*p* = 0.026), sugar (*p* = 0.008), SFA (*p* = 0.004), palmitic acid (*p* = 0.024), stearic acid (*p* = 0.005), and alcohol consumption (*p* = 0.001) in comparison with the control group. On the other hand, participants with T2D had a higher intake of total fiber and soluble fiber (*p* < 0.001), protein (*p* < 0.001), polyunsaturated fatty acid (PUFA) (*p* < 0.001), omega 3 (*p* < 0.001), omega 6 (*p* < 0.001), linoleic acid (*p* < 0.001), α-linolenic acid (*p* = 0.002), eicosapentaenoic acid (EPA) (*p* = 0.003), docosahexaenoic acid (DHA) (*p* = 0.004), water (*p* = 0.003), and all vitamins (*p* < 0.001; except for retinol and thiamine), carotenoids (*p* < 0.001), and minerals (*p* < 0.001; except for calcium, sodium, and selenium).

### 3.2. Factors Related with the Dietary Pattern

**Figure 1:**
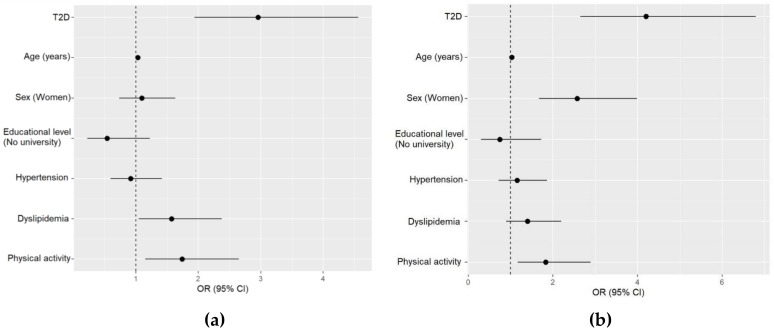
Multivariable analyses for the alternate Mediterranean Diet (aMED) score and alternate Healthy Eating Index (aHEI), including all the study groups. (**a**) Multivariable logistic regression for the aMED high tertile (6–9 points). Hosmer–Lemeshow test *p* < 0.001; (**b**) multivariable logistic regression for the aHEI high tertile (≥46 points). Hosmer–Lemeshow test *p* < 0.001. T2D, type 2 diabetes.

The multivariable analysis for the aMED score including all study participants revealed that T2D (OR: 2.96, 95% CI: 1.94–4.56; *p <* 0.001), increasing age (OR: 1.03, 95% CI: 1.01–1.05; *p =* 0.006), presence of dyslipidemia (OR: 1.57, 95% CI: 1.04–2.37; *p* = 0.031), and increased regular physical activity (OR: 1.74, 95% CI: 1.15–2.65; *p =* 0.009) were positively associated with high aMED score, after adjusting for potential confounders (Figure 1a). Furthermore, in the multivariable logistic analysis for the aHEI, T2D (OR 4.20, 95% CI 2.65–6.79; *p* < 0.001), increasing age (OR: 1.03, 95% CI: 1.00–1.05; *p =* 0.030), female gender (OR: 2.57, 95% CI: 1.67–3.98; *p <* 0.001), and increased physical activity (OR: 1.83, 95% CI: 1.17–2.89; *p* = 0.009) were positively associated with high aHEI score (Figure 1b).

### 3.3. Factors Related with the Dietary Pattern in Type 2 Diabetes

**Figure 2:**
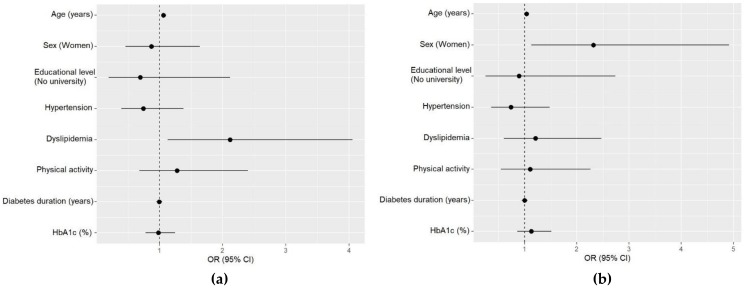
Multivariable analyses for the alternate Mediterranean Diet (aMED) score and alternate Healthy Eating Index (aHEI) of participants with type 2 diabetes. (**a**) Multivariable logistic regression for the high tertile (6–9 points) of the aMED. Hosmer–Lemeshow test *p* < 0.001; (**b**) multivariable logistic regression for the high tertile (≥46 points) of the aHEI. Hosmer–Lemeshow test *p* < 0.001.

The adjusted multivariable analysis including only the T2D group showed that increasing age (OR: 1.06, 95% CI: 1.03–1.11, *p* < 0.001) and dyslipidemia (OR: 2.12, 95% CI: 1.13–4.06, *p* = 0.021) were positively associated with the highest tertile of the aMED score (Figure 2a). On the other hand, the multivariable analysis for the aHEI showed that female gender (OR: 2.32, 95% CI: 1.13–4.92, *p* = 0.025) was related with higher aHEI scores in T2D (Figure 2b). We did not find any association between HbA1c or BMI with aMED and aHEI (data not shown).

## 4. Discussion

Our findings show that participants with T2D had a healthier dietary pattern with higher aMED and aHEI scores in comparison with a group without diabetes. High scores for both dietary quality indexes were positively associated with T2D, increasing age, and the practice of physical activity. Furthermore, the aMED score was also associated with the presence of dyslipidemia, while the aHEI score was related with female gender. In participants with T2D, age, and dyslipidemia were associated with higher aMED score and female gender was related with higher aHEI score.

Participants with T2D showed a higher adherence to the MedDiet and a healthier eating pattern compared with the control group; this is in contrast with the findings of a cross-sectional study performed on a small sample of participants in Ireland (*n* = 65 with T2D and *n* = 46 controls) [9]. However, apart from the small number of subjects, this study showed a great deal of missing data on the nutritional and dietary intake data that could have influenced the final results. Furthermore, in contrast with our results, the study by Vidal-Peracho et al., also conducted in Spain, found that subjects without diabetes had a higher adherence to the MedDiet in comparison with a group with both types of diabetes (type 1 and type 2) [10]. The analysis, including a group of both types of diabetes, also could affect the results due to the remarkable differences in the characteristics between T1D and T2D. Furthermore, our results were also discordant with those of Huffman et al. [8]; they found a lower aHEI in Haitian American and African American participants with T2D in comparison with a control group even though participants with T2D had a higher HEI. However, the aHEI is an alternative score adapted from the original HEI and is a better predictor of chronic disease risk due to a stronger inverse association with cardiovascular risk in comparison with the original HEI [18]. On the other hand, our results showed that the control group had a poorer adherence to the MedDiet; these findings are consistent with previous studies performed on large samples of healthy Spanish participants such as the EPIC Spanish cohort (*n* = 41,078), the SUN study (*n* = 13,609) and the ENRICA study (*n* = 12,948) [33,34,35].

In our Mediterranean country, participants with T2D receive MNT focused on the MedDiet pattern and healthy eating. This could explain the association between the higher tertiles of aMED and aHEI with the presence of T2D. Moreover, increasing age and physical activity were associated with higher aMED and aHEI scores; this is similar to a prospective study performed with a large sample of women without T2D that found a positive association between aMED and aHEI with increasing age and physical activity [36]. Furthermore, the Nurse’s Health Study also found that a higher aHEI was related with regular physical activity in women [37]. However, our results indicate that the presence of dyslipidemia was associated with a higher aMED score. Scientific studies published about this issue found that the MedDiet reduces the risk of T2D improving postprandial lipemia, lipid profile, and cardiovascular risk factors [3,4,38,39,40,41,42,43]. Our results could be due to the fact that participants with dyslipidemia may have received specific dietary counselling based on the MedDiet to improve their lipid profile. Finally, female gender was related with a high aHEI; this is in line with previous studies that observed a healthier eating in women [13,36,37]. In contrast, other cross-sectional studies did not find differences between both sexes [15,16], and Vidal-Peracho et al. found that men had higher MedDiet scores in comparison with women [10]. The fact that gender was only associated with aHEI may be explained by the differences on how each of the indexes used, aMED and aHEI, measure the contribution of each of the components to the final score; actually, the lack of gender association with aMED may be mainly explained by the difference in how they assess alcohol consumption, i.e., aHEI scores alcohol intake, taking into account the different recommended alcohol consumption between men and women.

This study has some limitations. First, the cross-sectional study design did not allow us to establish a causal relationship between variables and study outcomes. Furthermore, this was a sub-study of previous studies, mainly designed to assess the quality of life and treatment satisfaction of participants with T2D [20], and the prevalence of prediabetes [21]. Although these findings may reflect the educational dietary intervention received from healthcare professionals, we cannot establish a direct link, as we did not assess the specific knowledge about the dietary recommendations in participants with diabetes. Furthermore, the relationship between higher adherence to the MedDiet and healthy eating with the presence of type 2 diabetes could be, at least, partly explained by a social desirability bias; however, there was no association of higher adherence to a healthy eating pattern and factors could be related to social desirability, like lower BMI or improved HbA1c. However, this study was performed with a large and well-defined sample of participants. The use of this FFQ can estimate the nutrient intake and food consumption of the previous five-year period from the visit with a good reproducibility of the dietary habits [25,26]. Furthermore, this is the first study designed to assess the differences in the dietary pattern (i.e., MedDiet and healthy eating) of the participants with T2D in comparison with a control group from the same population. Therefore, these results can be potentially important to establish new approaches for MNT in people with diabetes.

## 5. Conclusions

Participants with T2D had a higher adherence to the MedDiet and a healthier eating pattern in comparison with a group without diabetes. Further research is needed to establish a causal relationship and definitive conclusions about the effectiveness of MNT in this population.

## Figures and Tables

**Table 1:** Characteristics of the study groups.

Variables	T2D (*n* = 238)	Controls (*n* = 238)	*p* ^1^
Age (years)	57.1 (9.4)	56.9 (10.0)	0.790
Sex (women)	122 (51.3)	115 (48.3)	0.582
Educational level			0.149
Non-university level	217 (91.2)	226 (95.0)	
Graduate or higher	21 (8.8)	12 (5.0)	
Tobacco exposure	127 (53.6)	117 (49.2)	0.382
Regular physical activity	118 (49.8)	167 (70.5)	<0.001
BMI (kg/m^2^)	30.6 [28.1; 35.1]	26.3 [24.5; 28.8]	<0.001
Waist circumference (cm)	105.0 [98.0; 113.0]	97.0 [91.0; 104.0]	<0.001
Hypertension	128 (53.8)	62 (26.1)	<0.001
Dyslipidemia	113 (47.5)	77 (32.4)	0.001
Diabetes duration (years)	10.1 (8.7)	-	-
HbA1c (%)	7.5 [6.8; 8.5]	5.6 [5.4; 5.9]	<0.001
HbA1c (mmol/mol)	58.0 [51.0; 69.0]	37.7 [35.5; 41.0]	<0.001
Total cholesterol (mg/dL)	182.0 [162.0; 211.0]	208.0 [183.0; 234.0]	<0.001
HDL-cholesterol (mg/dL)	49.0 [42.0; 58.0]	55.0 [48.0; 67.0]	<0.001
LDL-cholesterol (mg/dL)	106.0 [87.6;128.0]	129.0 [108.0; 150.0]	<0.001
Triglycerides (mg/dL)	118.0 [85.0; 171.0]	98.0 [74.0; 134.0]	<0.001
Diabetes therapy			<0.001
Diet	31 (13.0)	-	
OAD	134 (56.3)	-	
OAD + insulin	55 (23.1)	-	
Insulin	18 (7.6)	-	

Data are shown as *n* (*%*) or median [interquartile range]. ^1^ *p* was calculated according to the method of Benjamini and Hochberg for multiple comparisons. BMI, body mass index; HbA1c, glycated hemoglobin; HDL-cholesterol, high density lipoprotein-cholesterol; LDL-cholesterol, low density lipoprotein-cholesterol; OAD, oral antidiabetic agents; T2D, type 2 diabetes; Tobacco exposure, current and former smokers.

**Table 2:** Dietary quality index of the study groups.

Variables	T2D (*n* = 238)	Controls (*n* = 238)	*p* ^1^
aMED	4.3 (1.5)	3.5 (1.8)	<0.001
aMED (tertiles)			<0.001
T1 (0–3)	72 (30.3)	128 (53.8)	
T2 (4–5)	114 (47.9)	83 (34.9)	
T3 (6–9)	52 (21.8)	27 (11.3)	
aHEI	43.9 (6.5)	39.4 (7.4)	<0.001
aHEI (tertiles)			<0.001
T1 (20–38)	48 (20.2)	117 (49.2)	
T2 (39–45)	95 (39.9)	75 (31.5)	
T3 (46–64)	95 (39.9)	46 (19.3)	

Data are shown as *n* (%) for tertiles and as mean (SD) for continuous variables. ^1^ *p* was calculated according to the method of Benjamini and Hochberg for multiple comparisons. aMED, alternate Mediterranean Diet score; aHEI, alternate Healthy Eating Index; T2D, type 2 diabetes.

## Appendix A

**Table A1:** Daily food consumption of the study groups.

Food Groups (g/Day) ^1^	T2D (*n* = 238)	Controls (*n* = 238)	*p* ^2^
Dairy products	375.0 (254.0)	301.0 (198.0)	0.011
Eggs	19.8 (12.0)	22.4 (16.6)	1.000
White meat	47.0 (42.5)	34.9 (18.6)	0.002
Red meat	57.4 (40.0)	57.3 (33.2)	1.000
Processed meat	35.9 (26.8)	32.9 (24.9)	1.000
Meat	140.0 (61.3)	125.0 (45.3)	0.077
Lean fish	39.3 (29.0)	30.0 (22.0)	0.002
Fatty fish	41.3 (65.0)	29.8 (47.2)	0.738
Seafood	12.7 (11.7)	12.7 (12.4)	1.000
Fish	93.2 (82.3)	72.5 (57.0)	0.039
Fruits and vegetables	641.0 (228.0)	468.0 (192.0)	<0.001
Nuts	10.9 (15.8)	8.7 (9.2)	1.000
Legumes	33.2 (40.1)	24.2 (21.4)	0.062
Cereals and pasta	56.6 (44.8)	68.3 (39.0)	0.067
Potatoes	62.6 (42.5)	58.1 (62.7)	1.000
Bread	108.0 (63.1)	130.0 (76.5)	0.016
Sweets	33.9 (43.1)	37.6 (32.5)	1.000
Vegetable fat	23.0 (12.5)	27.3 (18.0)	0.080
Animal fat	0.8 (3.3)	0.2 (0.5)	0.234
Alcohol drinks	102.0 (172.0)	143.0 (184.0)	0.341
Non-alcoholic beverages	1421.0 (610.0)	1241.0 (486.0)	0.011
Coffee and tea	336.0 (322.0)	380.0 (281.0)	1.000
Prepared meals	107.0 (115.0)	76.7 (55.6)	0.009
Salt	0.1 (0.3)	0.4 (0.5)	<0.001

Data are shown as mean (SD). ^1^ Adjusted for energy intake. ^2^ *p* was calculated according to the method of Benjamini and Hochberg. T2D, type 2 diabetes.

**Table A2:** Daily nutrient intake of the study groups.

Nutrient Intake (Units/Day) ^1^	T2D (*n* = 238)	Controls (*n* = 238)	*p* ^2^
Energy intake (Kcal)	2149.0 (604.0)	2153.0 (587.0)	0.942
Glycemic load (%)	94.6 (19.9)	99.0 (22.5)	0.034
Carbohydrate (g)	216.0 (36.0)	214.0 (37.6)	0.668
Complex carbohydrate (g)	90.2 (20.0)	94.7 (21.0)	0.026
Sugar (g)	88.6 (27.0)	81.4 (28.8)	0.008
Added sugar (g)	20.3 (17.5)	25.7 (23.5)	0.008
Fiber (g)	26.8 (5.3)	23.6 (5.4)	<0.001
Soluble fiber (g)	4.3 (1.2)	3.6 (1.0)	<0.001
Insoluble fiber (g)	14.9 (4.5)	14.3 (4.6)	0.225
Protein (g)	104.0 (19.6)	96.7 (16.7)	<0.001
Total fat (g)	87.9 (13.6)	87.3 (15.4)	0.668
SFA (g)	23.2 (4.3)	24.5 (5.4)	0.004
MUFA (g)	42.2 (9.7)	42.0 (10.9)	0.858
PUFA (g)	15.9 (4.9)	14.3 (3.0)	<0.001
Omega 3 (g)	1.8 (0.6)	1.5 (0.6)	<0.001
Omega 6 (g)	14.0 (4.9)	12.6 (2.9)	<0.001
Trans fat (g)	1.0 (0.7)	1.1 (0.5)	0.058
Cholesterol (mg)	325.0 (202.0)	307.0 (108.0)	0.279
Palmitic acid (16:0) (g)	14.1 (2.2)	14.6 (2.6)	0.024
Stearic acid (18:0) (g)	5.5 (1.2)	5.9 (1.4)	0.005
Oleic acid (18:1ω-9) (g)	39.8 (9.4)	39.6 (10.7)	0.858
Linoleic acid (18:2ω-9) (g)	13.9 (4.9)	12.5 (2.9)	<0.001
α-linolenic acid (18:3ω-9) (g)	1.1 (0.2)	1.1 (0.2)	0.002
Arachidonic acid (20:4ω-6) (g)	0.2 (0.1)	0.2 (0.1)	0.366
EPA (20:5ω-3) (g)	0.2 (0.2)	0.2 (0.2)	0.003
DHA (22:6ω-3) (g)	0.4 (0.3)	0.3 (0.3)	0.004
Alcohol (g)	6.9 (12.5)	12.6 (21.2)	0.001
Caffeine (g)	186.0 (220.0)	203.0 (219.0)	0.431
Water (g)	2955.0 (798.0)	2745.0 (613.0)	0.003
Vitamin A (µg)	1472.0 (698.0)	1157.0 (646.0)	<0.001
Retinol (µg)	342.0 (430.0)	364.0 (491.0)	0.656
Carotene (µg)	1127.0 (592.0)	780.0 (387.0)	<0.001
α carotene (µg)	830.0 (682.0)	534.0 (462.0)	<0.001
β carotene (µg)	6128.0 (3236.0)	4254.0 (2078.0)	<0.001
β cryptoxanthin (µg)	422.0 (230.0)	292.0 (159.0)	<0.001
Lutein+zeoxanthin (µg)	5391.0 (4528.0)	3870.0 (2320.0)	<0.001
Lycopene (µg)	4479.0 (2312.0)	3925.0 (2326.0)	0.014
Folate (µg)	337.0 (74.2)	274.0 (61.0)	<0.001
Vitamin B_12_ (mg)	9.8 (4.1)	8.7 (4.3)	0.007
Vitamin B_6_ (mg)	2.2 (0.6)	1.9 (0.4)	<0.001
Vitamin C (mg)	161.0 (66.4)	108.0 (47.4)	<0.001
Vitamin D (mg)	4.3 (2.1)	3.8 (1.6)	0.002
Vitamin E (mg)	13.7 (3.6)	11.5 (3.1)	<0.001
Thiamine (mg)	1.7 (0.2)	1.6 (0.3)	0.087
Riboflavin (mg)	2.3 (0.5)	2.1 (0.5)	0.005
Niacin (mg)	29.4 (7.3)	27.2 (6.2)	0.001
Niacin equivalents (mg)	46.6 (10.4)	42.7 (8.4)	<0.001
Calcium (mg)	1101.0 (278.0)	1047.0 (299.0)	0.058
Iron (mg)	14.8 (2.6)	13.7 (2.5)	<0.001
Sodium (mg)	3314.0 (623.0)	3254.0 (652.0)	0.363
Potassium (mg)	3842.0 (672.0)	3281.0 (576.0)	<0.001
Magnesium (mg)	436.0 (86.3)	390.0 (75.1)	<0.001
Zinc (mg)	12.2 (1.9)	11.6 (1.9)	0.001
Selenium (µg)	151.0 (35.5)	145.0 (29.5)	0.091

Data are shown as mean (SD). ^1^ Adjusted for energy intake. ^2^ *p* was calculated according to the method of Benjamini and Hochberg. EPA, eicosapentaenoic acid; DHA, docosahexaenoic acid; MUFA, monounsaturated fatty acid; PUFA, polyunsaturated fatty acid; SFA, saturated fatty acid; T2D, type 2 diabetes.
